# How often do you cheat? Dispositional influences and intrapersonal stability of dishonest behavior

**DOI:** 10.3389/fpsyg.2024.1297058

**Published:** 2024-06-20

**Authors:** Kai Leisge, Christian Kaczmarek, Sabine Schaefer

**Affiliations:** Institute of Sport Sciences, Saarland University, Saarbrücken, Germany

**Keywords:** dishonesty, lying, cheating, deviation, psychological factors, intrapersonal stability, gender differences

## Abstract

Dishonesty, including lying, cheating, deception, and deviating from societal norms, has far-reaching implications across various aspects of modern society. From minor consequences like social discontent to severe outcomes such as economic damage through tax evasion, dishonest behavior affects us in multiple ways. This study investigates whether gender and psychological traits contribute to dishonest behavior, and whether unethical conduct is stable across diverse tasks. We examined 63 participants using a “Difference Spotting Task” (DST) and two motor tasks (1. coordinative throwing; 2. isometric strength). Dishonesty was measured by comparing self-reported performance with actual performance, allowing for a comprehensive analysis of both occurrence and extent of dishonesty. Our findings indicate that gender does not significantly influence the occurrence or extent of dishonest behavior. Moreover, we discovered that “Social Desirability” positively influences the extent of dishonesty, while “Task Orientation” increases the likelihood of engaging in dishonest acts. The study also reveals that the level of dishonesty remains relatively stable across all three tasks at an intrapersonal level.

## 1 Introduction

Social interactions in everyday life form the basis of human society, whether through verbal or non-verbal communication at work/education or in leisure activities such as sport. In order to maintain a functioning society, it is important that organizations and individuals follow certain rules or base their behavioral decisions on norms and moral values. Despite potential consequences for violating norms and moral values, such behavior is widespread, with approximately one-third of daily conversations involving deception or lies (Burgoon et al., 2003; van Kleef et al., 2015). These tendencies are also observed in sports, leading to distortions in competition, doping, corruption within associations, and fan violence (Frenger and Pitsch, 2021). A recent systematic review indicated doping prevalence rates in competitive sports ranging from 0 to 73% (Gleaves et al., 2021), with ~10% of recreational athletes in Europe engaging in the improper use of over-the-counter medication to enhance sporting performance (Christiansen et al., 2023). While minor norm deviations may cause no damage other than social discontent, major deviations in the form of bribery, doping, and tax evasion can lead to serious economic damage (Loewen et al., 2013).

The objective of this study is to identify factors influencing dishonesty in an experimental setting. Our paradigm allows us to investigate whether dishonest behavior shows variability or intrapersonal stability across different tasks.

Lies, deception and dishonest behavior come in many forms, and various terms are often used interchangeably to describe them. The following paragraphs outline the various contexts associated with dishonesty and introduce measures used to identify dishonest behavior. Two prominent theoretical perspectives, namely economic and social psychology (self-licensing), are discussed, providing insights into the interplay between material gain and self-perception. Additionally, the sociological approach introduces the concept of anomie by referring to Opp's specification (Opp, 1974; Lamnek, 2021). Combining these theories leads the way for formulating the hypothesis related to intrapersonal stability. The introduction continues by addressing gender and achievement motivation as factors potentially influencing dishonest behavior (DB), exploring various perspectives drawn from previous empirical studies.

A large and growing body of experimental research only addresses debates about who behaves dishonestly and under what circumstances (Fischbacher and Föllmi-Heusi, 2013; Clot et al., 2014; Ezquerra et al., 2018; Waeber, 2021). The following questions often remain unanswered: Why do humans cheat? What factors drive dishonesty? Is a person's tendency to behave dishonestly stable across different tasks or scenarios (intrapersonal stability; Muñoz García et al., 2021)? Understanding the determinants of dishonest behavior is crucial. It is essential for establishing ethical norms, implementing effective prevention strategies in different contexts, and shaping legislation and policies (Jacobsen et al., 2018). This understanding plays a key role in fostering integrity and trust both inside and outside of organizations (Kindsiko et al., 2013; LaDuke, 2013) and guides educational practices (Hodgkinson et al., 2016), while further advancing research in social psychology and human behavior. However, since dishonest behavior primarily revolves around violating rules or norms, its replication in an experimental set-up can be challenging (Jacobsen et al., 2018). People often try to conceal this type of behavior or conform to social desirability, leading to the use of a relatively heterogeneous set of experimental tasks (Gerlach et al., 2019).

Data can either be collected at an aggregate or individual level. Prominent examples of measuring dishonesty at an aggregate level include “*coin-flip*” tasks (Chowdhury et al., 2021) or “*die-roll*” tasks (Grosch and Rau, 2017). In these tasks, participants can cheat to “win” by misreporting a randomly generated outcome (coin-flip or die-roll) that is not observable for the experimenter. The rate of dishonesty can only be estimated by comparing the aggregated reported “win” outcomes of a sufficiently large sample to the theoretical/statistical baseline distributions of expected “wins” (Rosenbaum et al., 2014). On the one hand, this may allow for anonymity, as individuals do not have to fear detection (Fischbacher and Föllmi-Heusi, 2013). On the other hand, it also makes it impossible to link specific personality traits to dishonesty. Commonly used measures to identify dishonesty at an individual level include ability tests, such as the *matrix* task (Mazar et al., 2008), where self-reported outcomes are compared to the actual performances, deception games, or unsolvable paradigms. In unsolvable paradigms, participants are asked to indicate whether they solved a specific task, even though the tasks are designed to be unsolvable (Liu et al., 2021). Examples such as “*sender-receiver*” games or “*tax compliance*” experiments are utilized to detect deceptive behavior in either interactive and non-interactive communication settings (Burgoon and Buller, 1994; Capraro, 2018). Despite some drawbacks (see Heyman et al., 2020; Liu et al., 2021 or Blume et al., 2002 for further information), these approaches allow for direct inferences about individual behavior, making them essential for accurately linking personal factors to dishonesty. One of the rarely discussed drawbacks of tasks like the *matrix* task is the occurrence of *honest mistakes* (Heyman et al., 2020). This term refers to the tendency for many small lies to actually be genuine errors. Since the *matrix* task heavily relies on the participants' mathematical abilities, it is particularly prone to these *honest mistakes*. Participants may unknowingly miscalculate and believe they have solved an unsolvable matrix, resulting in an honest error rather than a deliberate falsehood (Heyman et al., 2020).

### 1.1 Why do people engage in dishonest behavior?

When discussing the question as to why people display DB, two theories rooted in economics and social psychology are mentioned repeatedly. The economic perspective (*homo economicus*) can be described as a model of rational and selfish human behavior, where the expected external benefits are weighed against the costs of DB (Henrich et al., 2001; Mazar et al., 2008). Humans therefore would only show DB if the material incentives, in the sense of wealth maximizing, outweigh those of acting honestly (Becker, 1968; Rosenbaum et al., 2014; Gerlach et al., 2019). However, subjects are often reluctant to deviate to the maximum extent, which does not speak in favor of a solely economic approach (Mazar et al., 2008; Rosenbaum et al., 2014; Ezquerra et al., 2018; Abeler et al., 2019). A complementary perspective is given by the self-licensing theory. It is based on internalized norms of honesty and the existence of an intrinsic cost of DB (Rosenbaum et al., 2014). People are either (a) the ethical type and are unwilling to perform DB regardless of the benefit, or (b) a mixed type who appear to have a finite positive intrinsic cost of DB, and finally (c) the economic type who have a zero cost of DB (Kajackaite and Gneezy, 2017). Considering both of these perspectives, one might argue that people not only consider the material gain expected from DB, but also how this behavior influences their self-perception (Waeber, 2021). When linking these two theories to the aforementioned research question regarding intrapersonal stability or variability, a definitive direction cannot be unequivocally determined. Rather, it is reasonable to hypothesize that both intra- and interpersonal differences in DB may exist. While individuals categorized as the ethical or economical type demonstrate intrapersonal stability concerning conforming or dishonest behavior, participants classified as the mixed type exhibit behavioral variability across tasks.

Another theoretical access, rarely discussed in experimental research of DB, is provided by sociology. The basic idea of the anomie theory by Emil Durkheim is that a lack of ethical norms or social standards can cause a state of normlessness, resulting in uncertainty regarding the consequences of individual behavior (Lamnek, 2021). An extension and specification of this theory was introduced by Karl-Dieter Opp. He investigates the determinants and variables that could influence DB. The first variable he mentions is the “*intensity of goals internalized by the individual*,” which measures the degree to which a person desires these achievements (Opp, 1968). The adaptation of these internalized goals may differ depending on the individual, their self-concept, and the specific experimental task. Participants for whom achieving a high performance in one of the tasks is more important than in the other two, will have varying goal intensities which can influence dishonest behavior. A second variable is given by the “*intensity of illegitimate norms*” and describes the acceptance of socially defined illegitimate means (Opp, 1974). Inter-individual differences among participants, influenced by socialization and cultural background, cannot be ruled out and may therefore impact DB. Lastly the “*degree of illegitimate means and opportunities*,” is also essential when trying to explain DB (Simmler et al., 2017). To minimize the impact of this factor, the opportunities to engage in dishonest behavior should be equal for all participants in all conditions and tests. According to Opp, higher levels of these three factors favor the development of DB. This is contrasted by the variables “*intensity of legitimate norms*” and the “*degree of legitimate means and opportunities*,” which focus on the beliefs that certain goals can be achieved legally and the extent to which individuals believe they can reach their goals by following legitimate paths (Opp, 1968, 1974; Lamnek, 2021). A higher degree of these two determinants reduces the likelihood of DB. The “*intensity of legitimate norms*” can also vary between individuals due to factors such as socialization processes or cultural distinctions. If the aspect “*degree of legitimate means and opportunities*” is attributed not only to situational contextual factors but also to the individual capabilities of each participant in the respective task, this may also influence DB. For instance, subjects with higher strength abilities have more pronounced means and opportunities to perform well in the corresponding task and thus achieve higher financial gain without being dishonest. If this assumption holds true, negative correlations should be found between the observed performance of individuals and the extent of DB.

According to this explanation, it would seem that DB is not only influenced by the trade-offs between external and internal resources, like the economic and social psychology approaches would suggest. It is rather a complex balancing act of internalized goals and the acceptance and extent of legitimate and illegitimate means. The question arises if this balancing act is internally stable for individuals (intrapersonal stability), or if it is being adapted for different tasks or scenarios (intrapersonal variability). Therefore, the following hypothesis is formulated:

H_1_: *Participants will show behavioral stability of DB throughout the three tasks*.

Participants were told the goal of this study was to investigate cognitive learning processes within sensory (visual) perception. Contrary to the assumption made by Opp's Anomie Theory that high performance in various tasks leads to an increased “*degree of legitimate means and opportunities*,” Liu et al. (2021) found evidence suggesting that this may not be the case for visual search ability. Due to these conflicting positions, this relationship will be further examined in the following exploratory hypothesis.

H_2_: *Dishonest behavior will be more prevalent among individuals with high performances in a visual search task*.

### 1.2 Gender as an influencing factor for dishonest behavior

Gender may influence DB, but it is crucial to note that previous empirical studies are not conclusive. Several studies demonstrated that male participants show a higher level of DB than females (Grym and Liljander, 2016; Gerlach et al., 2019; Kennedy and Kray, 2022). For example, Grosch and Rau (2017) tested 268 participants in a “*die-roll*” task, similar to the approach by Fischbacher and Föllmi-Heusi (2013), and report a greater extent of DB for males compared to females. Waeber (2021) also reports that women are more honest on average than men in a task involving self-reported outcomes that influence their financial pay-out. Previous findings on deception in “*sender-receiver*” games (Dreber and Johannesson, 2008) or in a social interaction setting with face-to-face communication (Lohse and Qari, 2021) also lend support to this direction. Chowdhury et al. (2021) present different results depending on specific experimental instructions. This finding is consistent with other studies that have also found no gender differences (DePaulo et al., 1996; Aoki et al., 2010; Childs, 2012). For example, Ezquerra et al. (2018) also used the “*die-roll*” task to detect DB. They report that males and females do not cheat differently and therefore show DB to a similar extent. Additional results on deceptive behavior indicate that there are no gender differences observed in a “*sender-receiver*” game (Gylfason et al., 2013) or in a “*tax compliance*” experiment (Lohse and Qari, 2014). Few studies also suggest a higher amount of dishonesty or deception for women compared to men in a “die-roll” task (Clot et al., 2014; Ruffle and Tobol, 2014), or a deceptive communication experiment (Tyler and Feldman, 2004). Potential explanations for gender differences in DB include the prevalence of prosocial individuals (Grosch and Rau, 2017), the question of whether dishonest behavior could be planned (Chowdhury et al., 2021), cultural differences among the respective samples (Aoki et al., 2010; Childs, 2012), or highly specific samples and operationalizations of dishonesty (Ruffle and Tobol, 2014).

Given the heterogeneity of these results, a novel approach should be employed. Such an approach should allow for the reliable and repeated detection, at an individual level and across different tasks, not only of the mere existence of DB (in the following described as the binary variable “*frequency*” with the two levels “*dishonest*” and “*honest*”) but also of its exact amount (referred to as the metric variable “*extent*” with different levels depending on the task). Based on varied evidence of DB in both males and females, and acknowledging that the differences found are contingent upon various factors, we plan to analyse gender differences in an exploratory way without formulating directional hypotheses.

H_3_: *The extent of DB will be different for males compared to females*.

H_4_: *The number of participants showing DB will be different in males compared to females*.

### 1.3 Achievement motivation as an influencing factor

To further explain DB, it is important to consider other psychological determinants. One frequently discussed theory is based on the individual's achievement motivation (Nicholls, 1989). People with a higher focus on task orientation tend to use self-referential criteria and subjective success, whereas people with a higher focus on ego/goal orientation define their success in relation to others (Duda et al., 1991). The manifestation of these two orientations, in turn, influences the intensity of goals internalized by the individual, as specified in Karl-Dieter Opp's formulation of the Anomie Theory (Opp, 1974; Lamnek, 2021). The recent literature only suggests that a high task orientation should be positively correlated with general moral values, and high ego orientation should lead to inappropriate behavior (Kavussanu and Ntoumanis, 2003; Gonçalves et al., 2010). To date, only few other studies have examined the relationship between different achievement motivations and DB. Lucidi et al. (2017) did not find a significant correlation between task- or ego-orientation and the observed cheating in competitive tennis matches. Ring and Kavussanu (2018) used an experimental setting, where athletes could decide to illegitimately improve their race time to enhance their chances of winning. They reported a higher ego orientation in cheating individuals compared to honest ones. The operationalization and measurement of DB in these studies was based on the direct performance comparison with other individuals. Participants were only rewarded based on their performance relative to their opponents, such as winning a point in tennis or receiving a financial benefit for winning. This can impact the effect of achievement motivation. Hence, it is crucial to investigate whether the association between ego- or task-orientation and DB changes when the experimental tasks do not involve a direct performance comparison with other individuals and thus only self-referenced criteria for performance evaluation exist.

Since little is known about possible effects of other psychometric factors, explorative analyses of this study therefore also focus on the influence of Honesty-Humility (individuals with high scores avoid manipulating others for personal gain and feel little temptation to break the rules) and social desirability (tendency to conform to societal expectations) on DB, which has not received a lot of attention in previous research. It is proposed that:

H_5_: *Lower levels of Honesty-Humility and higher levels of Social Desirability, Task- and Ego- Orientation will lead to a higher extent of DB*.

H_6_: *Lower levels of Honesty-Humility and higher levels of Social Desirability, Task- and Ego-Orientation will lead to higher frequencies of DB*.

## 2 Materials and methods

### 2.1 Participants

A total of 65 students were tested. In terms of gender, the sample is almost balanced. It includes 31 (48%) men between the ages of 18 and 38 (*M* = 22, *SD* = 3) and 34 (52%) women between the ages of 18–31 (*M* = 21, *SD* = 2). All subjects were active in sports and engaged in either individual sports (*n* = 48; e.g., track and field, distance running, gymnastics, etc.) or team sports (*n* = 17; e.g., handball, volleyball, soccer, etc.). Participants had normal or corrected to normal (female: *n* = 14; male: *n* = 8) vision. Only one individual reported color blindness. Table 1 provides the age, sports activity, and psychometric measures for each gender group and for the entire sample.

**Table 1:** Summary statistics by gender.

	**Gender**			**Test statistics**
	**Male (*****n*** = **30)**	**Female (*****n*** = **33)**	**Total (*****n*** = **63)**	
**Age [years]**				
*M*	22.4	21.39	21.87	*z* = −1.919; *p* = 0.055
*SD*	3.17	2.81	3		
*MIN*	18	18	18		
*MAX*	35	31	35		
**Sport [min/wk]**				
*M*	484.33	376.67	427.94	*t* = −1.876; *p* = 0.066
*SD*	261.74	182.51	228.42		
*MIN*	120	90	90		
*MAX*	1,440	780	1,440		
**Task orientation**				
*M*	3.9	4.19	4.05	*z* = −1.129; *p* = 0.259
*SD*	0.64	0.47	0.57		
*MED*	4	4	4		
*MIN*	2.43	3.43	2.43		
*MAX*	4.71	5	5		
**Ego orientation**				
*M*	3.47	2.91	3.18	*t* = −2.670; ***p*** **=** 0.009^**^
*SD*	0.81	0.84	0.86		
*MED*	3.5	2.83	3.33		
*MIN*	2	1.67	1.67		
*MAX*	5	4.5	5		
**Social desirability**				
*M*	10.73	12.18	11.49	*t* = 1.984; *p* = 0.052
*SD*	2.79	3	2.97		
*MED*	11	13	12		
*MIN*	4	6	4		
*MAX*	15	17	17		
**Honesty humility**				
*M*	3.47	3.7	3.59	*t* = 1.682; *p* = 0.098
*SD*	0.57	0.51	0.54		
*MED*	3.35	3.67	3.6		
*MIN*	2.6	2.7	2.6		
*MAX*	4.5	4.6	4.6		

Values for the test statistics and the corresponding p-values are derived from two sample Welch's t-tests for sport [min/wk], ego-orientation, social desirability and honesty humility and from two sample Wilcoxon tests for age [years] and task-orientation. Significant values are highlighted in bold for visual emphasis. ^*^p < 0.05; ^**^p < 0.01.

All participants were recruited via self-selection from the participant pool of the Sport Science Institute at Saarland University, which mainly includes sport students and few psychology students. No specific inclusion or exclusion criteria were defined prior to the study. An e-mail with a short description of the study and a list of possible time slots was sent out 2 weeks before the 16-week testing period started. All testing sessions were conducted exclusively within the laboratory setting of the Sport Science Institute. Participants signed informed consent before the study began. To encourage active participation and to trigger DB, participants received different monetary rewards based on their performance in each task. The financial outcome ranged from 2.80 Euro to 9.01 Euro with a mean of 4.73 Euro (*SD* = 0.97). The study was approved by the Ethics Committee of Saarland University.

### 2.2 Measures and covariates

#### 2.2.1 Difference spotting

##### 2.2.1.1 Dishonest behavior

**Figure 1:**
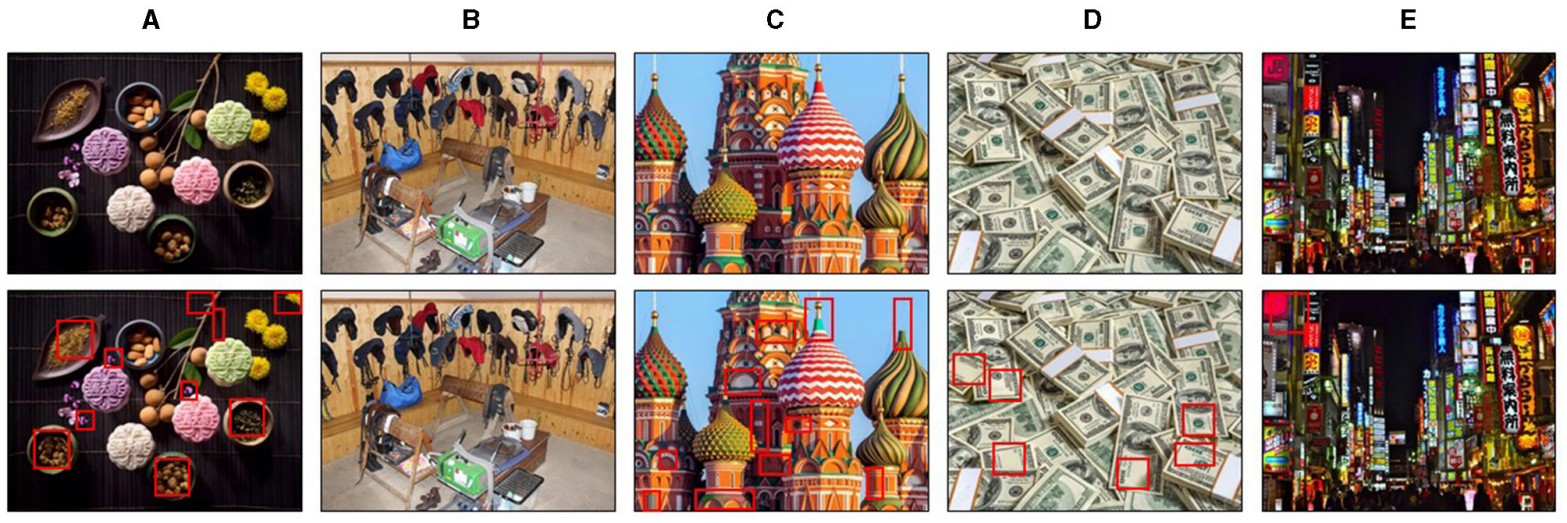
Examples of the visual stimuli used in this study. **(A)** Example of an original stimulus pair in “solvable items,” belonging to the category ”easy.” **(B)** Example of an original stimulus pair in “unsolvable items,” containing no differences. Participants were instructed that there would be two additional difficulty levels besides **(C)** “easy,” namely **(D)** “medium” with six differences, and **(E)** “hard” with one difference. Note, however, that the instructions differed from the actual stimulus pairs. Differences between the target stimuli are highlighted by red boxes for illustration purposes (Adapted from Liu et al., 2021).

The “Difference Spotting Task” (DST) is a new cognitive measure designed to assess dishonest behavior at the item and individual level. It was introduced by Liu et al. (2021), and is based on the “unsolvable items” paradigm. The authors took several measures to minimize the extent of *honest mistakes*. The DST is a non-verbal task that does not rely on language or mathematical skills, making it applicable to a wide population (Liu et al., 2021). In this computer version of the task, participants were asked to identify the differences between two similar but not identical pictures (Figures 1A, B). Contrary to the instruction, only 40 of the 80 pairs are solvable. In order to maintain the credibility of the test, subjects were told that each pair of pictures was of varying difficulty. While some items would belong to the category labeled “easy” and therefore contain 10 differences, other pairs would be categorized as “medium” with six differences, and finally the category “hard” would consist of images with only one difference (Figures 1C–E).

**Figure 2:**
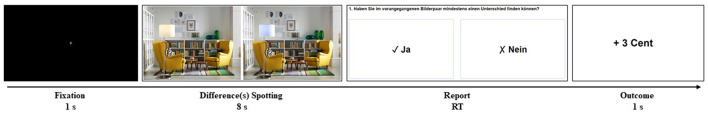
Sequence of events in a single trial in the DST, starting with fixation (1 s) and followed by a pair of pictures for 8 s. The subsequent “Report”-Screen was not timed. Based on the chosen answer (“Yes” or “No”) the corresponding outcome screen was shown (1 s). This trial shows that a participant answered “Yes” in a solvable item and therefore gained 3 cents (Adapted from Liu et al., 2021).

During the DST itself, participants only had to indicate whether they found a difference (“✓ Yes”) or not (“χ No”). There was no need to mark or point out the differences they found. Participants were instructed to double-check any differences they found and only select “yes” if they were certain. Figure 2 shows the timeline for a single trial. Due to the high cognitive demands of this task and to further minimize the occurrence of *honest mistakes*, it was decided to include a standardized break of 60 s after 40 trials. The DB can be measured as the sum of claimed-to-be-solved pairs within the 40 unsolvable items. Participants received 3 cents/solved pair in this task.

##### 2.2.1.2 Visual search ability

In this second computer task, participants were asked to not only report whether they found a difference or not, but also to actually mark the spotted differences. Starting again with a fixation screen for 1 s, each of the following total 10 trials lasted 30 s and consisted of a pair of pictures with exactly 10 differences. Remaining time was always shown on the same screen. Each trial ended with a specific and untimed transition screen that indicated the end of the trial. Participants could individually choose when to start the next trial. The overall score was presented on the last screen of this assessment. The instruction was to mark as many differences as possible. The participants could set a maximum of 10 different markers for this task. The correctness of the placed markers was standardized by programming areas around the actual differences. Care was taken to adjust the areas to the respective sizes of the differences. This ensured the objectivity of the evaluation. Individuals had no opportunity to engage in dishonest behavior in this task. As a measure for performance, the total sum of spotted differences across all 10 trials was used. Participants received 2 cents for each difference marked in this task.

#### 2.2.2 Motor performance

##### 2.2.2.1 Coordinative throwing

The experimental set-up consisted of a bucket (diameter = 28 cm; height = 32 cm) that was placed on the floor as a target. Participants were asked to stand behind a chair 3 m from the target. The main objective was to score as many hits as possible in five blocks of 10 throws each. A throw was counted as a hit if the ball (soft ball with 6 cm in diameter) touched the bottom of the bucket. Rim shots should not be registered as successful hits. To minimize the occurrence of *honest mistakes*, only 10 balls were available for throwing, and the target was designed so that successful hits remained in the bucket. This way, participants could easily recount their successful hits. They were asked to write down their performances via self-disclosure after each of the five blocks and received 5 cents for each successful hit.

##### 2.2.2.2 Isometric strength task

A bar was placed 2.40 m above the floor (distance to the wall = 40 cm). Participants were instructed to pull themselves up to the chin-up position, starting from a box (height = 40 cm). The main objective was to hold the chin-up position, with their head held over the bar, for as long as possible. Only one trial was used in this scenario because of muscle fatigue. Concerning the posture and position of their hands, individuals were free to grab the bar however they preferred. A laptop facing the participants and displaying a running timer, that was programmed to start at zero after a countdown of 20 s expired, was placed on a chair ~3 m away from them so that they could accurately measure their own performance. They were instructed to report the time when they left the chin-up position. Participant could earn 3 cents for every second in the target position.

#### 2.2.3 Questionnaires

##### 2.2.3.1 Achievement goals

The achievement goals of participants were assessed using the reliable and validated German version of the Task and Ego Orientation in Sport Questionnaire (TEOSQ-D), which was developed by Rethorst and Wehrmann (1998). The questionnaire starts with a common stem for each item (“I feel most successful in sport when …”) and is followed by 13 items measuring task (seven items) and ego orientation (six items) on five-point Likert scales ranging from strongly disagree (1) to strongly agree (5). The evaluation is done separately for the two subscales by determining the mean value. Higher scores on each subscale represent greater achievement goals.

##### 2.2.3.2 Honesty-humility

To get a measure for honesty, part of the German version of the HEXACO personality inventory was used (Ashton et al., 2014). The scale consists of the dimensions Honest-Humility (H), Emotionality (E), Extraversion (X), Agreeableness (A), Conscientiousness (C), and Openness to Experience (O). We only used the subscale of Honesty-Humility, because it is the only subscale for which a relationship with DB had been shown prior to the current study (Hershfield et al., 2012). It states that persons with a high score avoid manipulating others for personal gain and feel little temptation to break the rules. This subscale consists of 16-items that are answered on five-point Likert scales ranging from strongly disagree (1) to strongly agree (5).

##### 2.2.3.3 Social desirability

Social desirability was assessed by using the modified German version of the “Social Desirability Scale” originally published by Crowne and Marlowe (1960). The scale consists of 17 items with the response options “true” (1) and “false” (0). It has been shown to be a valid and reliable assessment (Stöber, 1999). Higher scores represent a greater level of social desirability.

### 2.3 Data collection

In the experiment, participants were informed that data would be anonymized and treated confidentially. Test sessions lasted ~60 min each and were conducted in an individual setting with only the experimenter and the participant being present. Each session started with subjects completing computer-based questionnaires providing information about gender, age, visual aid, sporting activity, achievement goals, Honesty-Humility and Social-Desirability. Thereafter participants proceeded to complete the DST and skill task on the same computer, where they received written instructions. Throughout these first two assessments, the experimenter remained in the same room, though not directly visible to the subjects. The experimenter was not able to see the computer screen of the participants. For the subsequent two tests (coordinative throwing and isometric strength), participants were informed that this phase of the experiment aimed to examine the influence of spectator effects on motor performance. However, as there were no spectators, each individual was told they were in the “*alone*” condition, where no one, including the experimenter, was present. This means that, after reading the standardized instructions to them, the experimenter exited the room, and participants were left alone to complete the task. A hidden camera was placed on target to measure the actual performance in these tasks. As a measure for DB the difference between the self-reported and the actual performance was used. A research assistant who did not know any of the students and is also not involved in teaching them evaluated the videos for both, the coordinative throwing task and the isometric strength task. As the study adopted a within-subjects design, each participant completed every task.

### 2.4 Statistics

The statistical analysis was conducted using R Statistical Software for Windows (R Core Team, 2022). Whether DB occurs for each of the tasks was tested using one sample *t*-test or equivalent non-parametric alternatives, by comparing the empirical values against 0. A linear mixed effects model, with participant as a random factor, and task as a within factor, was used to predict the influence of gender, Honesty-Humility, Social Desirability, Task- and Ego-Orientation on DB across all three *z*-standardized tasks. To predict the occurrence of DB, a logistic mixed effects model, with participant as a random factor, and task as a within factor, was performed using the same personal and psychometric variables. To assess whether the participants show intrapersonal variability or stability in DB, a new variable was derived from the data. For each individual, the sum of squared differences from their respective individual mean across the three standardized variables was computed, followed by a division by 3. Individuals whose resulting value approaches zero tend to demonstrate a higher degree of stability in their behavior. Conversely, higher values indicate a greater magnitude of intrapersonal variability. Intrapersonal variability of DB was tested using non-parametric one sample Wilcox test, by comparing the empirical values against 0.219, which would represent a difference in means with a small effect. This threshold was determined by using the formula for Cohen's *d*, substituting the respective values of *d, s*, μ_1_ and then solving for μ_2_ (Cohen, 1988). The mixed effects models were conducted with the nlme R package (Pinheiro et al., 2022). Descriptive statistics and Cronbach's α were calculated via the psych R package (Revelle, 2022). Effect sizes were computed by using the rcompanion R package (Mangiafico, 2023). For all analyses the alpha level was set to 0.05. Two participants were excluded from all analyses due to instruction non-compliance.

## 3 Results

### 3.1 General results: is dishonest behavior present in the three tasks?

In the DST 59% of participants (*n* = 37) reported finding a difference for at least one out of 40 unsolvable items, whereas only 14% (*n* = 9) claimed to have solved all 40 solvable pictures. Additionally, the performance, i.e., the amount of solvable and unsolvable items claimed to be solved by the participants, is shown in Table 2. Individuals reported solving 10% (*SD* = 15.99; *CV* = 159%) of unsolvable items on average, which is significantly more than 0% (Wilcoxon signed rank test, *z* = −5.428, *p* < 0.001, *r* = 0.873, representing a large effect). Reliability analysis revealed an excellent internal consistency (α = 0.94). Opposite to the findings of Liu et al. (2021) no significant relation between the performance in solvable and unsolvable pictures could be observed using spearman's rank correlation, *rho* = 0.016, *p* = 0.903. The analysis of the *skill task* also revealed that there is no significant difference between honest and dishonest individuals in the amount of differences they are able to spot [Welch two sample *t*-test, *t*_(60.926)_ = 0.139, *p* = 0.889]. This is in line with the results obtained in the linear model, where the extent of DB in the DST was predicted by the performance in the skill task. The model only explains a statistically non-significant and very weak proportion of variance, *R*^2^ < 0.001, *F*_(1, 61)_ = 0.001, *p* = 0.986.

**Table 2:** Number of reported differences for each task by gender and in total.

	**Gender**			**Test statistics**
	**Male (*****n*** = **30)**	**Female (*****n*** = **33)**	**Total (*****n*** = **63)**	
**DST—solvable**				
*M*	37.5	37.52	37.51	*z* = −0.622; *p* = 0.839
*SD*	1.94	1.75	1.83		
*MED*	38	38	38		
*MIN*	33	32	32		
*MAX*	40	40	40		
**DST—unsolvable**				
*M*	3.3	4.67	4.02	*z* = −0.203; *p* = 0.534
*SD*	5.21	7.33	6.4		
*MED*	1	2	1		
*MIN*	0	0	0		
*MAX*	21	28	28		
**Skill task**				
*M*	52.43	50.3	51.32	*t* = −0.936; *p* = 0.353
*SD*	8.08	9.95	9.1		
*MED*	51.5	51	51		
*MIN*	31	21	21		
*MAX*	69	65	69		

Values for the test statistics and the corresponding p-values are derived from a two sample Welch's t-test for the Skill-Task of spotting and marking differences and from two sample Wilcoxon tests for solvable and unsolvable blocks of the DST.

In the “*Throwing Task*” 49% of participants (*n* = 31) over reported their performance by at least 1 successful hit, ranging from 1 to 20 hits (*M* = 3.35; *SD* = 3.96; *CV* = 118%). Across all participants, we detected DB which is significantly larger than 0 (Wilcoxon signed rank test, *z* = −5.026, *p* < 0.001, *r* = 0.88, representing a large effect).

In the “*Strength Task*” 56% of participants (*n* = 35) over reported their performance by at least 1 s, ranging from 1 to 17 s (*M* = 4; *SD* = 4.46; *CV* = 103%). Across all participants, we detected DB which is significantly larger than 0 (Wilcoxon signed rank test, *z* = −5.316, *p* < 0.001, *r* = 0.879, representing a large effect).

### 3.2 Factors influencing the extent of dishonest behavior

We fitted a linear mixed effects model (estimated using maximum log-likelihood) to predict the extent of *z*-standardized DB scores with the within-subject factor task. The model included the identification variable (“Participant”) as a random effect. Its total explanatory power is moderate (conditional *R*^2^ = 0.24) and the part related to the fixed effects alone is small, *R*^2^ = 0.07. Within this model the effect of social desirability is statistically significant and positive [β = 0.071; 95% CI [0.01, 0.13], *t*_(125)_ = 2.4, *p* = 0.018], indicating that a higher amount of social desirability leads to a higher extent in *z*-standardized cheating scores. The fixed effects of gender, honesty-humility, task- and ego-orientation failed to reach significance (see Table 3). During the assessment of assumptions, it was ascertained that linearity might be problematic within the framework of this model (see Supplementary material). The model was subsequently examined with non-linear components, yielding no conclusive evidence of a non-linear relationship for the Social Desirability variable. As a result, the linear effects reported below are presented and will be discussed later.

**Table 3:** Results of the linear mixed effects model showing a significant influence of social desirability on the extent of dishonest behavior.

**Effect**	**Estimate**	**SE**	**95% CI**		***t***	***p***
			**LL**	**UL**		
Gender	0.217	0.185	−0.15	0.58	1.174	0.245
Social desirability	0.071	0.030	0.01	0.13	2.401	**0.018** ^*^
Ego orientation	−0.005	0.108	−0.22	0.21	−0.047	0.963
Task orientation	0.239	0.163	−0.08	0.56	1.472	0.144
Honesty humility	−0.099	0.160	−0.41	0.22	−0.619	0.539

Significant values are highlighted in bold for visual emphasis. ^*^p < 0.05; ^**^p < 0.01.

### 3.3 Factors influencing the occurrence of dishonest behavior

We fitted a logistic mixed effects model (estimated using maximum log-likelihood and Nelder-Mead optimizer) to predict the occurrence of DB with the within-subject factor task. The model included the identification variable (“Participant”) as a random effect. Its total explanatory power is small (conditional *R*^2^ = 0.11) and the part related to the fixed effects alone is also small, *R*^2^ = 0.09. Within this model the effect of task orientation is statistically significant and positive [β = 0.834, 95% CI [0.20, 1.47], *z* = 2.097, *p* = 0.010]. By analyzing the odds ratios, it can be observed that a one-unit increase in Task Orientation is associated with a 2.3-fold higher probability of showing DB. The fixed effect for gender, honesty-humility, ego orientation, and social desirability failed to reach significance (see Table 4).

**Table 4:** Results of the logistic mixed effects model showing a significant influence of Task-Orientation on the occurrence of dishonest behavior.

**Effect**	**Estimate**	**SE**	***z*-value**	***p***	**OR**	**95% CI**	
						**LL**	**UL**
Gender	0.431	0.348	1.238	0.216	1.539	0.778	3.047
Social desirability	0.097	0.058	1.674	0.094	1.102	0.983	1.236
Ego orientation	0.011	0.204	0.056	0.955	1.012	0.678	1.510
Task orientation	0.834	0.322	2.590	**0.010** ^*^	2.303	1.225	4.329
Honesty humility	−0.114	0.302	−0.376	0.707	0.892	0.493	1.614

Significant values are highlighted in bold for visual emphasis. ^*^p < 0.05; ^**^p < 0.01.

### 3.4 Intrapersonal stability

Combining all three tasks where DB could be measured, a total of eight individuals (13%; men: *n* = 4; women: *n* = 4) remained completely honest, 28 (44%; men: *n* = 11; women: *n* = 17) showed DB in exactly one task, 17 (27%; men: *n* = 9; women: *n* = 8) were dishonest in two of the three tasks, and 10 participants (16%; men: *n* = 6; women: *n* = 4) over reported their performance in every single task. These frequencies are not significantly different between men and women (chi-squared test, χ^2^ = 1.605, *p* = 0.658, ϕ_*c*_ = 0.159). While some information about the tendency for stability or variability of DB can be drawn from the frequencies listed above, an additional inferential approach is necessary to analyze the extent. Individuals reached a mean of 0.501 (*SD* = 1.097; CV = 219%) in intrapersonal variability, which is not significantly >0.219 (Wilcoxon signed rank test, *z* = −3.379, *p* = 0.705, *r* = −0.083, representing a weak effect), indicating that the extent of DB is stable across tasks.

## 4 Discussion

This experiment was designed to repeatedly measure DB on the individual level across two different motor and one cognitive task. Its goal was to investigate the intrapersonal stability of DB, and to further identify influencing factors. The study design allows for a direct assessment of DB for each individual, without calculating probabilities and comparing them to an expected distribution of a true random event (as in die-roll or coin-flip tasks). Therefore, to our knowledge, this experimental design may be the first to allow inferences from self-reported personality traits to the extent of observed DB measured in different tasks. The detailed measurement of DB additionally makes it possible to not only categorize participants into honest and dishonest individuals, but also to determine the exact extent of dishonesty.

### 4.1 Gender

Neither the proportion of participants showing DB, nor the extent of DB is significantly different between men and women in the models we calculated. Our hypothesis must therefore be rejected. These results align with the empirical studies, which also found no gender-related differences (Aoki et al., 2010; Childs, 2012; Pascual-Ezama et al., 2015; Ezquerra et al., 2018). This might be explained with the observations of Chowdhury et al. (2021) who propose vanishing gender differences when pre-planning of dishonest actions becomes possible. Participants in their study were aware of the two tasks and could pre-plan their behavior, or they learned about the individual task in each stage and were not able to plan accordingly. While the exact definition of pre-planning remains unclear, we argue that participants in our study were also instructed beforehand and therefore had a chance to pre-plan their behavior. This might explain the non-significant difference in the occurrence of DB in both men and women.

In conclusion, reference will once again be made to Opp's specification of the Anomie Theory. Since no gender differences were found, it can be assumed that either one or a combination of the “*intensity of internalized goals*” and the “*intensity of legitimate and illegitimate means*” is similarly manifested for both men and women across all three tasks. The assumed variability in participants' self-concept does not appear to exert a significant enough influence on the intensity of these objectives to provoke gender differences in DB to a substantial degree.

### 4.2 The influence of psychometric factors

#### 4.2.1 Social desirability

The mixed model analysis indicates that higher social desirability leads to more extensive DB. As the extent of DB has not been previously linked to psychometric variables, the results reported here should be regarded as novel exploratory findings that require validation in future research. However, the analysis of the logistic model examining the occurrence of DB fails to strengthen these findings. A greater amount of social desirability does not translate into a higher likelihood of showing DB.

#### 4.2.2 Task orientation

The logistic mixed model analysis indicates a significant relationship between task orientation and the occurrence of dishonest behavior. Individuals with a stronger inclination toward task orientation demonstrate DB with a notably heightened likelihood. This complements both the theoretical assumptions of Kavussanu and Ntoumanis (2003) and Gonçalves et al. (2010). However, the empirical findings of Ring and Kavussanu (2018) only report a significant influence of ego orientation on DB, while task orientation had no impact. The reason for these divergent results may lie in the employed task. In the investigation by Ring and Kavussanu (2018), subjects undertook a competitive task that entailed a direct performance comparison with others within the experimental setting. As a result, the extent of the reward was intricately tied to this competitive context. In our study however, no opportunity for a performance comparison between individuals was possible. The financial reward solely depended on individual performance. In Ring and Kavussanu (2018) study, the task consisted of outperforming other participants, while our approach made participants' pursue self-referenced performance criteria. Consequently, it is plausible that in the tasks utilized in our study, DB was shown solely from individuals with higher levels of task orientation. Due to the absence of a direct performance comparison and the subsequent lack of an externally referenced definition of success, individuals with higher Ego Orientation were maybe not prompted enough to engage in DB. It seems that in line with the theoretical assumptions of the Anomie Theory by Opp, the intensity of goals internalized by the individual and consequently the likelihood to engage in DB are influenced differently by the manifestations of ego and task orientations.

#### 4.2.3 Honesty humility

The study conducted here presents a novel approach in this realm of research. As such it cannot be definitively ascertained why a significant impact of the psychometric variable Honesty-Humility on the extent or occurrence of DB could not be observed. In accordance with the construct definition of this particular subscale, an aspect to be measured is the propensity for “temptation to break the rules.” However, honest and dishonest participants do not differ in the manifestation of this subscale. Nevertheless, it is plausible that the presence of socially desirable response tendencies could exert an influence on the applicability of the scale to real-world contexts.

### 4.3 Intrapersonal stability

By analyzing the frequency of DB (i.e., categorization of whether participants were honest or dishonest), the experimental design employed in this study provides empirical evidence for the validation of the typologies introduced within the theoretical framework of the self-licensing theory. Those who showed intrapersonal stability remained completely honest (ethical type), or were dishonest in every single task (economical type). Such behavior can be referred to as Behavioral Spillover (Chowdhury et al., 2021). It describes consistent behavior across repeated decision-making scenarios, implying that an individual who behaves honestly (dishonestly) should continue such actions in recurring opportunities (Chowdhury et al., 2021). However, this behavioral pattern cannot be generalized to all participants. The vast majority of participants changed their behavior at least once between the tasks and can be categorized as the mixed type with a finite positive intrinsic cost of DB. By obeying the rules in one task, participants with intrapersonal variability in DB earn moral credit that reduces the discomfort of performing dishonestly afterwards (moral licensing; Clot et al., 2014; Chowdhury et al., 2021). However, actions can also be oriented in the opposite direction. The moral self-worth of individuals can be restored through moral actions that can balance their inner moral account.

The subsequent inferential statistical analysis of the extent of DB (i.e., the exact difference between the self-reported and actual observed outcome) revealed that participants show intrapersonal behavioral stability. The variable computed using an index does not exhibit a significant increase beyond the mean difference indicative of a small effect. This constitutes a novel finding concerning the extent of DB, which is situated within the theoretical paradigms of Opp's Anomie Theory (Opp, 1974), the self-licensing theory, and the concept of Behavioral Spillover. By recognizing that the level of dishonest behavior remains consistent across participants, we can draw conclusions about expected behavior. For example, if individuals consistently misreport whether a tennis ball touched the line, resulting in a loss of points, it suggests a likelihood of similar behavior in the future. This principle can be extended to broader contexts, such as doping, dishonesty in academic settings, and organizational environments. Once such dishonest individuals are identified, targeted interventions can be implemented to reinforce factors that promote honesty, such as the perceived “*intensity of legitimate norms*” and the perceived “*degree of legitimate means and opportunities*” from Opp's anomie theory. Future studies should try to repeatedly measure DB across more similar tasks, in order to prove if the task itself has an influence on intrapersonal stability of dishonesty.

### 4.4 Limitations

#### 4.4.1 Limitations concerning the lack of a gender effect

A possible reason discussed in empirical studies of gender differences in dishonesty is the presence of country- and cultural specific norms (Childs, 2012; Rosenbaum et al., 2014). For example, there might be less of a difference between male and female German sport students than between Australian men and women (Friesen and Gangadharan, 2012). Norms and moral values vary across cultures due to their acquisition within distinct social environments. According to the 6-D model of national culture, societies differ in six dimensions: Individualism; Power Distance; Masculinity; Uncertainty Avoidance; Long-term Orientation; and Indulgence (Hofstede et al., 2010). Therefore, categorizations of dishonesty and conformity could vary across different cultures and introduce social biases into these studies. Given that our study did not explore cultural dimensions, we cannot draw any firm conclusions, and we suggest to include cultural differences in future studies on DB. While Fischbacher and Föllmi-Heusi (2013) argue that cheating outcomes remain the same even when the stakes are tripled or the anonymity is altered, Muñoz García et al. (2021) show that gender differences in DB are influenced by the reward factor. Women are more satisfied with lower rewards and would therefore need higher rewards to trigger DB (Muñoz García et al., 2021). Given the absence of a gender difference in DB, it can be argued that rewards were high enough to trigger dishonesty in both men and women in our sample. Given that the financial payoff was based on the original study by Liu et al. (2021), future studies should aim to determine the threshold at which women's DB is triggered.

Grosch and Rau (2017) mention that prosocial individuals are most honest and that the highest percentage of these subjects can be found among women. Another plausible explanation for the lack of significant gender differences could stem from variations in the expression of prosociality. Male participants within the study's sample might exhibit a heightened degree of social value orientation, or female participants might display a lower degree compared to the general population. Future studies should aim to determine if there are different thresholds at which men's DB and women's DB are triggered.

Given the variety of different experimental tasks and methodological differences between the studies, we suggest that the influence of gender on DB may be more complex than previously assumed. We argue that the differences in methodology and the operationalization of DB are the key reasons for inconsistent results.

#### 4.4.2 Limitations concerning intrapersonal stability of dishonest behavior

A factor contributing to the intrapersonal variability of behavior can be highlighted within the context of Opp's Anomie Theory (Opp, 1974). It is plausible that among certain participants, the intensity of goals internalized may vary from task to task, consequently inducing or inhibiting DB accordingly. While it remains unclear why the tasks trigger DB differently, it should be noted that some participants might not value one of those tasks equally to the others. In other words, motivations for participation, success and DB may vary from task to task. The employed experimental design did not yield quantitative data that could directly quantify or characterize the “intensity of goals internalized by the individual.” Future studies should try to operationalize this variable throughout different tasks, for example by asking participants before working on a task how motivated they are to solve it, or how much they like this type of tasks in general.

#### 4.4.3 General limitations

It is possible that the experimenter could exert an influence on participants' DB through impression management biases. It remains questionable to compare results of DB across samples with a high degree of variability without taking the additional factors mentioned above into account. While the extent and occurrence of DB might not be different, it is still possible that men and women may differ in subtler aspects like type of incentive to induce dishonesty (Childs, 2012).

Furthermore, this study solely examined whether dishonest behavior can be induced among participants through the provision of material rewards. It is important to note that the monetary compensation might hold diverse implications for individuals as well. Although our experimental design minimizes the problem of honest mistakes (Heyman et al., 2020; Liu et al., 2021) we cannot exclude the possibility that participants occasionally perceived a difference (in the DST) where there was none. Similarly, they may have miscounted the successful hits in the throwing task without intending to cheat.

Additionally, the high specificity of the sample should be acknowledged. Given that the participants primarily consist of sports students, the generalizability of the findings to the broader population is questionable.

## 5 Conclusion

In conclusion, the present study has provided valuable insights into the influencing factors and intrapersonal stability of DB. While both males and females show DB in all three tasks, no difference in the frequency or the extent could be observed between them. The extent of DB is stable across individuals and it is further influenced by social desirability. Task orientated subjects show a higher likelihood of the mere occurrence of DB. These findings make significant contributions to our understanding of the potential determinants of DB and illuminate the application of Opp's Anomie Theory. They reveal that dishonest behavior is not solely driven by the desire to maximize certain outcomes but is also influenced by other factors, such as the “*intensity of goals internalized by the individual*” and the “*intensity of illegitimate norms*.” Additionally, experimentally proving the existence of the “*ethical*,” “*mixed*,” and “*economic types*” could further strengthen the self-licensing theory.

## Data availability statement

The raw data supporting the conclusions of this article will be made available by the authors, without undue reservation.

## Ethics statement

The studies involving humans were approved by Ethics Committee for Empirical Human Sciences of Saarland University. The studies were conducted in accordance with the local legislation and institutional requirements. The participants provided their written informed consent to participate in this study.

## Author contributions

KL: Conceptualization, Data curation, Formal analysis, Investigation, Methodology, Writing – original draft, Writing – review & editing. CK: Conceptualization, Methodology, Writing – review & editing. SS: Conceptualization, Methodology, Project administration, Writing – review & editing.
