# Supplementary material for: How often do you cheat? Dispositional influences and intrapersonal stability of dishonest behavior
Source: Front Psychol. 2024 Jun 20;15:1297058. doi: 10.3389/fpsyg.2024.1297058 (PMC11222642; doi:10.3389/fpsyg.2024.1297058)
Supplement: Supplementary file 1 [file Table_1.DOCX]

Supplementary Material

How often do you cheat? Dispositional influences and intrapersonal stability of dishonest behavior

Kai Leisge*^1^, Christian Kaczmarek^1^ and Sabine Schäfer^1^

^1^University of Saarland, Sportwissenschaftliches Institut, Saarbrücken, Germany

*** Correspondence:**Kai Leisge
kai.leisge@uni-saarland.de

# Supplementary Figure


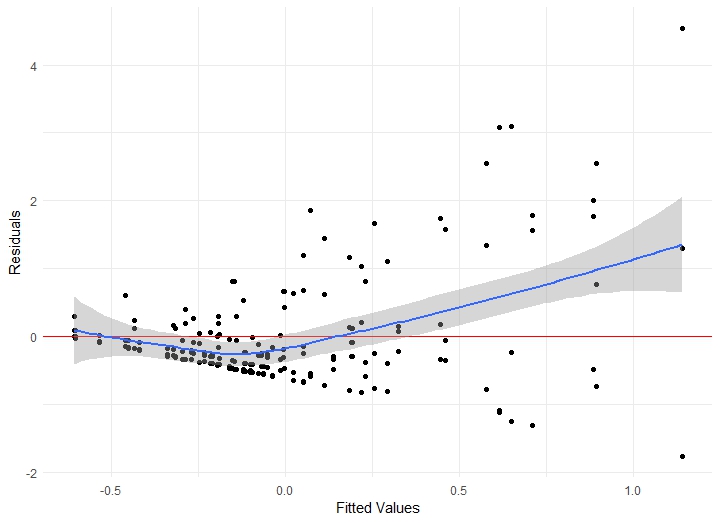


**Supplementary Figure 1.** Plot of the model residuals and the observed fitted values within the linear mixed effects model to check for the assumption of linearity.
